# A preliminary study of aged care facility staff indicates limitations in awareness of the link between depression and physical morbidity

**DOI:** 10.1186/1471-2318-13-30

**Published:** 2013-04-09

**Authors:** Joanna Atkins, Sharon L Naismith, Georgina M Luscombe, Ian B Hickie

**Affiliations:** 1 Brain & Mind Research Institute, University of Sydney, 94 Mallett Street, Camperdown, NSW, 2050, Australia; 2 Clinical Research Unit, Brain & Mind Research Institute, University of Sydney, Sydney, Australia; 3 Department of Obstetrics & Gynaecology, Sydney Medical School, University of Sydney, Sydney, Australia; 4 Brain & Mind Research Institute, University of Sydney, Sydney, Australia

**Keywords:** Elderly, Depression, Physical illness

## Abstract

**Background:**

It is important to understand the complex inter-relationship between depression and physical illness in order to plan and provide quality health care services for older persons and reduce suffering and early mortality. This study assessed the awareness and knowledge of age-care staff of the link between physical morbidity and depression.

**Methods:**

One hundred and nineteen staff from both residential (high and low care) and community aged care facilities were surveyed on their awareness and knowledge of the relationship between physical morbidity and symptoms of depression. Predictors of levels of knowledge were assessed using multiple regression analysis.

**Results:**

Awareness of the link between physical morbidity and symptoms of depression was generally high. However, while nearly eighty percent of respondents said they had had training in mental health, they were only able to answer an average of six out of ten of the knowledge questions correctly. Predictors of knowledge were: higher age, higher educational status and working in a high care facility.

**Conclusions:**

Responses to the survey questions demonstrated gaps in knowledge about the relationship between depression and physical health. The need for regular ongoing training to improve knowledge and awareness of this relationship is indicated. Treatment of physical health issues which is essential in reducing the risk for depression in older persons in aged care environments could be optimized by improved staff training.

## Background

The link between depression and physical illness has been well documented e.g. [1]. Older persons residing in aged care facilities are particularly likely to have high rates of physical illness and disability as well as high rates of depression. A recent Australian Bureau of Statistics report showed that of those older persons living in care facilities, 97% had some form of disability, and of these, 91% had a physical disability, 72% sensory or speech disabilities, 57% psychological disabilities and 31% had disabilities related to head injury, stroke or brain damage [2]. An Australian study found approximately half of high care residents and a third of low care residents were depressed [3]. This compares to rates of depression between 1 to 16% for older persons living in the community [4-7]. The relationship between depression and physical morbidity is bidirectional and appears attributable to depressive disorders precipitating and prolonging physical morbidity and physical morbidity exacerbating and prolonging depression [1,8] leading to a cycle of poor physical and mental health. It is important for age-care staff to understand the complex inter-relationship between depression and physical illness so they can identify care recipients who may be at risk and provide appropriate evidence-based interventions in a timely manner to avoid unnecessary suffering.

A number of studies have demonstrated the association between depression and physical morbidity in general age groups. For example, one study [9] found that the likelihood of depression increased with the increasing number of physical health conditions. Other studies have shown that patients with depression are more likely to develop coronary artery disease [10], to have a stroke [11], or a heart attack [12] and the relationship between depression and pain has been documented, see [13] for a review. There is less research examining the link between depression and physical illness specifically in elderly populations but a relationship has been found between depression and cardiovascular and cerebrovascular disease [14], osteoporosis and psychiatric illness [15,16], and depression and diabetes [17]. A cross sectional study [18] found that both physical comorbidities and functional limitations increased the likelihood of depressive illness in older persons and a meta-analysis found that chronic illness was a risk factor for depression in this age group [19]. The relationship between depression and functional impairment or mobility disability in elderly populations has also been established e.g. [20-22].

Depression also adversely affects survival rates and outcomes for many of these disorders including cancer [23,24]; stroke [25]; asthma [26]; diabetes [27] and quadruples the risk of mortality in the months following a heart attack [28].

There is evidence that treating the physical condition can improve depression and treating depression can improve physical morbidity. For example, studies have shown that antidepressant treatment of post-stroke depression decreases depression, helps recovery of cognitive function and increases survival rates [29,30]; cardiac rehabilitation has been shown to improve depressive symptoms [31]; good management of arthritis can lead to reductions in fatigue, anxiety, pain and depression [32]; and improved depression care in those with arthritis improves pain, functional status and quality of life [33]. A collaborative care treatment model improved both depression and functional status in older patients with diabetes [34]. It is therefore essential that all health conditions (physical and psychological) are appropriately treated to give the care recipient the best possible outcome.

While a number of studies have examined the knowledge of depression held by various health care professionals [35-38], and age-care staff [39-41], none of these asked about the relationship between depression and physical health factors. To our knowledge, only one published study has explored awareness of the link between depression and physical health, which is the specific area of interest of the current study. Blumenfield et al. [42] examined the public’s awareness of the link between depression and cardiac health in a sample of 559 US adults aged between 40 and 69 years. They found that 86% of respondents recognized that depression could affect physical health, 30% were aware that depression was a risk factor for cardiac disease and 32% were aware that depression increased the risk for having a second heart attack.

Given the link between depression and physical illness, it is important for age-care staff to recognize this relationship in order to facilitate treatment for both conditions and provide optimum care for care recipients to reduce mortality and morbidity. Thus the current study aims to assess the awareness and knowledge of age-care staff of the depression/physical illness link. There does not appear to be any previous published research that looks specifically at the awareness and knowledge of age-care staff regarding this association. It is hypothesized that professional staff (e.g. managers and nurses) will have greater awareness and knowledge of the link due to higher levels of training than non-professional staff (personal care workers), that those with a higher educational level will also have greater awareness and knowledge. It is also predicted that staff from high care facilities will have greater awareness and knowledge than those from low care and community care facilities because of their greater exposure to care recipients with multiple physical health issues. The current study also examines whether staff factors, specifically length of service, age, gender or training in mental health predict levels of awareness and knowledge.

## Method

### Sample

The sample consisted of 119 staff (including personal carers, nurses, managers and other professionals) from high and low care residential facilities, and from community care organisations. In Australia, care recipients are assigned to high or low care status in accordance with the Aged Care Funding Instrument (ACFI) guidelines based on the severity of scores on three domains: activities of daily living, behaviour, and complex health care (see:http://www.health.gov.au/internet/main/publishing.nsf/Content/ageing-acfi-using-weightings.htm). Nursing home accommodation is provided for care recipients with high support needs and hostel accommodation for care recipients with low support needs who do not have complex ongoing care needs but require some assistance with the demands of daily living. In community care in Australia, visiting direct care workers provide needs-based care to older persons with both high and low support needs, in their own homes, including personal care, cleaning, shopping etc. and is similar to the care provided by personal care assistants in residential facilities. For this reason care staff from both residential care facilities and from community care organizations are considered ‘personal carers’ in the current study.

### Measures

A survey was devised by the authors (published researchers in the field of older age and depression: including one psychiatrist, one psychologist and one neuropsychologist) to examine awareness and knowledge of the relationship between physical morbidity and depression and included:

Demographic factors

Age, gender, occupation, length of time employed in current position, length of time employed in the aged care sector, training in mental health and education status (no formal education; completed or partially completed primary school; completed or partially completed early high school; completed or partially completed senior high school; certificate or diploma; degree; postgraduate diploma, Masters or PhD).

Awareness questions

Twenty-three items assessed the awareness of staff of the link between physical health factors and symptoms of depression. All of these areas have been empirically shown to have bidirectional relationships with depression (see Background section of this report). Specifically, staff were asked to rate the degree to which they thought that the following broad groups of physical health factors: *poorer physical health*, *increased pain*, and *decreased mobility* led to the following five symptoms of depression: *low mood or feeling depressed*, *low energy/fatigue*, *difficulty sleeping*, *feeling anxious/tense*, *poor memory/concentration.* One additional symptom was added to the link with decreased mobility*: increased pain.* A fourth question examined awareness of the link in reverse and asked staff members to assess the extent to which they thought *low mood or feeling depressed* led to: *low energy/fatigue*, *difficulty sleeping*, *feeling anxious/tense*, *poor memory/concentration, increased problems with physical health*, *decreased mobility* and *increased pain.* Staff were asked to rate the extent that the one factor led to the other factors by placing a mark on a visual analog scale with three descriptive anchors such that a mark to the left indicated ‘a little’, around the middle of the scale indicated ‘a moderate amount’ and towards the right indicated ‘a great deal’. Responses were then given a score out of 10 depending on whereabouts on the line the respondent had placed their mark (i.e. distance from the left hand end of the line). These individual scores were totalled for all the questions giving a final score out of 230. Awareness of the relationship between depression and the other factors was operationalized as being indicated by scores on this scale, with a higher score indicative of greater awareness of the relationship.

Knowledge questions

Ten items assessed staff knowledge about the link between depression and specific physical disorders and disabilities. Staff were asked to indicate whether they believed a number of statements about the relationship between depression and particular physical health issues were true or false. The physical health issues included: *functional impairment, asthma, stroke, arthritis, heart disease and diabetes* and were all based on the findings of various studies of chronic disease and depression reviewed in [1] and agreed upon by consensus of the authors. Half of the ten statements were worded to be true and half false. For each correct response respondents were given a score of 1 which when totalled gave a final knowledge score out of 10.

### Statistical analysis

Analyses were conducted using SPSS Version 17. Staff care role was recoded into the dichotomous variable: *professional staff* (consisting of nurses, managers and other professionals) and *personal carers.* Demographic differences between the two groups of staff were assessed using independent samples t tests for continuous variables and chi-square analyses for categorical variables. Awareness and knowledge scores were compared between professional staff and personal carers and where there were demographic differences they were accounted for by performing an analysis of covariance. Overall differences in awareness and knowledge scores between the three types of care (high, low, community) were assessed using analyses of variance with post hoc Bonferroni contrasts for pairwise comparisons. Relationships between continuous variables (e.g. *level of knowledge* and *age*) were assessed using Pearson’s Correlation Coefficients. Univariate linear regression analyses were conducted with the outcome variables of *level of awareness* and *level of knowledge* and the predictor variables of *age, gender, role, length of service in current position, length of service overall, training in mental health, educational status,* and *type of care*. Education was recoded into the dichotomous categories: *up to certificate or diploma* versus *tertiary education*; and type of care was recoded into the dummy variables: *high care* versus *all other care types*, *low care* versus *all other care types* and *community care* versus *all other care types.* Where more than one predictor emerged as significant in the univariate regression, a multiple linear regression analysis was used to determine the relative contribution of the significant variables. This analysis used a multiple linear regression model with forced entry of *age* in block 1 (method = enter), followed by the significant predictor variables in block 2 (method = stepwise). Where relevant, analyses were two tailed and a significance level was set at 0.05 for all analyses.

### Ethics approval

Ethics approval to conduct the study was given by the University of Sydney Human Research Ethics Committee. Participants’ written informed consent was obtained.

## Results

### Demographics

As shown in Table 1, the majority of staff were female (89.1%) with an average age of 47.6 years (range: 21 to 67 years) and had achieved an educational level of up to certificate/diploma level (80.3%). Staff had been in the current position for an average of 5.0 years, in the aged care sector for an average of 8.3 years and 78.8% reported having had training in mental health. The majority of staff surveyed were personal carers (73.1%), while the remainder were: managers (11.8%), nurses (8.4%), and other professionals (6.7%).

**Table 1:** Demographic characteristics of the sample presented by type of role (n = 119)

		**Role type**		
		**Professional**	**Carers**	**Professional Vs Carers**
	N	(n = 32)	(n = 87)	p
Age (years), mean (SD)	114	50.3 (8.0)	46.5 (10.5)	0.043
Gender (female), % (n)	119	90.6 (29)	88.5 (77)	NS
Education (up to certificate/diploma), % (n)	117	75.0 (24)	82.4 (70)	NS
Length of service in current post (years), mean (SD)	117	6.1 (5.1)	4.6 (3.1)	NS
Length of service in aged care sector (years), mean (SD)	116	12.1 (9.1)	6.9 (6.1)	0.005
Trained in mental health, % (n)	118	78.1 (25)	79.1 (68)	NS
Type of care, % (n)	119			
High care		31.3 (10)	6.9 (6)	<0.001
Low care		46.9 (15)	24.1 (21)	
Community care		21.9 (7)	69.0 (60)	

NS, not significant

### Awareness questions

Staff were most likely to recognize the relationship between *increased pain* and *feeling sad, down or miserable* (receiving an average rating of 7.7 out of 10, SD 2.2), *increased pain* and *difficulty sleeping* (7.7, SD 2.2), *decreased mobility* and *feeling sad, down or miserable* (7.6, SD 2.0), *increased pain* and *decreased energy/fatigue* (7.5, SD 2.0), *poorer physical health* and *decreased energy/fatigue* (7.4, SD 2.0) and *low mood or feeling depressed* and *decreased energy/fatigue* (7.4, SD 2.2; see Table 2). Lowest awareness occurred for the relationship between *decreased mobility* and *poor memory/concentration* (mean 6.2, SD 2.3), *poorer physical health* and *poor memory/concentration* (6.4, SD 2.4), *decreased mobility* and *difficulty sleeping* (6.5, SD 2.3) and *low mood or feeling depressed* and *increased pain* (6.6, SD 2.4).

**Table 2:** Staff awareness of the relationship between depression and physical health factors

	**n**	**Mean score**^**†**^**(SD)**
**Q1. To what extent does poorer physical health lead to:**		
Feeling sad, down or miserable	118	7.1 (2.1)
Decreased energy/fatigue	118	7.4 (2.0)
Difficulty sleeping	118	6.9 (2.1)
Feeling anxious/tense	117	6.9 (2.2)
Poor memory/concentration	119	6.4 (2.4)
**Q2. To what extent does increased pain lead to:**		
Feeling sad, down or miserable	118	7.7 (2.2)
Decreased energy/fatigue	119	7.5 (2.0)
Difficulty sleeping	114	7.7 (2.2)
Feeling anxious/tense	114	7.3 (2.2)
Poor memory/concentration	115	6.7 (2.4)
**Q3. To what extent does decreased mobility lead to:**		
Feeling sad, down or miserable	115	7.6 (2.0)
Decreased energy/fatigue	116	7.2 (2.0)
Difficulty sleeping	115	6.5 (2.3)
Feeling anxious/tense	113	7.1 (2.0)
Poor memory/concentration	115	6.2 (2.3)
Increased pain	115	7.0 (2.3)
**Q4. To what extent does low mood or feeling depressed lead to:**		
Decreased energy/fatigue	118	7.4 (2.2)
Difficulty sleeping	119	7.0 (2.4)
Feeling anxious/tense	118	7.3 (2.4)
Poor memory/concentration	117	7.1 (2.3)
Increased problems with physical health	119	7.1 (2.2)
Decreased mobility	115	6.7 (2.3)
Increased pain	115	6.6 (2.4)

^†^out of 10, higher scores indicate greater awareness.

Professional staff had a mean total awareness score of 170.2 (SD 54.0) out of a possible 230 and personal carers a mean score of 159.1 (SD 34.9), however this difference was not significant. There were no significant differences in awareness scores for *type of care* (high care: mean 185.0, SD 39.4; low care: 155.4, 42.5; community care: 160.2, 40.4), *educational level* (lower education: 160.9, 39.8; higher education 160.9, 42.9), *training in mental health* (no training: 146.4, 38.9; training: 164.7, 40.2), or *gender* (male: 157.0, 43.0; female: 162.4, 40.3). There were no significant correlations between awareness score and either *age* or *length of service* (either in current position or in the aged care sector).

### Knowledge questions

Staff answered on average, six out of the ten knowledge questions correctly. As shown in Table 3, the questions that staff were most likely to answer correctly were those relating to depression and arthritis (management of arthritis: 82.1%, exercise: 79.3%), antidepressants and stroke (71.4%) and functional impairment and depression (69.2%). Staff were least likely to correctly answer questions relating to depression and stroke (depression as a risk factor for stroke: 35.4%, increased morbidity: 40.5%) and depression and diabetes (44.3%).

**Table 3:** Staff responses to knowledge questions about the relationship between depression and various physical health issues

		**Role Type**		
		**Professional**	**Carers**	**Professional Vs Carers**
	**N**	**Correct**	**Correct**	**p**
		**% (n)**	**% (n)**	
**True questions**:				
Depression is a leading cause of functional impairment in the elderly	117	87.5 (28)	62.4 (53)	0.009
People with diabetes are **more** likely to be depressed	115	46.9 (15)	43.4 (36)	NS
Persons with depression are **more** likely to have a stroke	113	34.4 (11)	35.8 (29)	NS
Good management of arthritis leads to reductions in fatigue, anxiety, pain and depression	117	87.5 (28)	80.0 (68)	NS
Persons who have depression following a stroke are **more** likely to die within two years	111	53.1 (17)	35.4 (28)	NS
**False questions**:				
Antidepressants given to depressed persons following a stroke **decrease** survival rates	112	68.8 (22)	72.5 (58)	NS
Persons who are depressed are **no** more likely to develop heart disease than persons who are not depressed	116	65.6 (21)	54.8 (46)	NS
Cardiac rehabilitation does **not** improve depressive symptoms	117	68.8 (22)	56.5 (48)	NS
People with asthma are **no** more likely to suffer from depression than people without asthma	116	50.0 (16)	60.7 (51)	NS
Exercise has been found to **increase** depression and pain in persons with arthritis	116	78.1 (25)	79.8 (67)	NS

NS, not significant.

Professional staff (mean 6.7, SD 1.5) answered more questions correctly than personal carers (5.9, 1.8; P = 0.017). However, after accounting for *age* and *length of service*, this difference was no longer significant. There was an overall significant difference in knowledge scores for the three types of care (F (2,107) = 505, P = 0.008) with post hoc Bonferroni contrasts revealing that the difference was between *high care* and *community care* (P = 0.007). Those with an educational status of *degree and higher* also answered more questions correctly (mean 7.1, SD 1.6) than those with an educational status *up to certificate/diploma level* (5.8, SD 1.6, P = 0.001). *Age* was significantly related to level of knowledge (r = 0.23, P = 0.018). *Length of service* (either in current position or in the aged care sector) was not related to level of knowledge. There were no other significant demographic differences.

### Univariate and multivariate analysis

In order to determine the most pertinent predictors of the two outcome variables: level of awareness and level of knowledge of the depression/physical health link, univariate analyses were conducted. Table 4 displays the univariate associations between the outcome variables and the potential predictor variables (*age*, *gender*, *role*, *length of service in current role*, *total length of service in aged care sector*, *training in mental health*, *educational status*, and *type of care dummy variables*). Five variables were significantly associated with level of knowledge – *age*, *role*, *educational status* and *high care* versus *other care types* and *community care* versus *other care types*. Only one variable was associated with awareness scores – *high care* versus *other types of care*. The significant predictors of level of knowledge were then entered into a multiple regression model. As shown in Table 5, after forced entry of *age*, the other factors shown to have a significant univariate association were subjected to stepwise elimination. *Age, educational status* and *high care versus other care types* were significant predictors, uniquely accounting for 4.2%, 9.5% and 6.2% of the variance respectively. Overall these variables accounted for 22.1% of the variance in knowledge scores (F (df = 3, 99) = 9.4, P < 0.001).

**Table 4:** Univariate linear regression analyses for level of awareness and level of knowledge

	**Level of awareness**	**Level of knowledge**
	**p**	**p**
Age	NS	0.018
Gender	NS	NS
Role	NS	0.003
Length of service (current position)	NS	NS
Length of service (in aged care)	NS	NS
Training in mental health	NS	NS
Education	NS	0.001
High care versus other care types	0.026	0.005
Low care versus other care types	NS	NS
Community care versus other care types	NS	0.015

NS, not significant.

**Table 5:** Multivariate linear regression analysis for level of knowledge

	**Beta weight**	**t**	**Unique r**^**2**^	**P**
**Block 1**: forced entry variable				
Age	0.204	2.3	4.2	0.024
**Block 2**: predictor variables				
Role	NS	NS	NS	NS
Educational level	0.310	3.5	9.5	0.001
High care/other care types	−0.250	−2.8	6.2	0.006
Community care/other care types	NS	NS	NS	NS
	**Total R**^**2**^	**F**	**P**	
	22.1	9.4	<0.001	

NS, Not significant.

## Discussion

Responses to the survey questions demonstrated gaps in knowledge about the relationship between depression and physical health, particularly in relationship to depression and stroke and depression and diabetes. The main predictors of knowledge of the mental/physical health link were higher age, higher educational status and working in a high care facility.

Only working in high care versus the other care types was predictive of level of awareness of the depression/physical health link. However, in regards to level of knowledge, a number of factors were found to be significant. Professional staff did appear to have greater knowledge of the relationship between depression and physical illness as hypothesized but this difference disappears when age and experience are taken into consideration. Similarly, higher educational status and being exposed to those with higher care needs (working in a high care facility) were related to increased knowledge of the depression/physical health link as predicted. Length of service was not related to either increased awareness or increased knowledge suggesting that greater experience on its own is not sufficient to increase awareness or knowledge.

These results highlight the importance of education and good quality training for those who work closely with older persons. It is especially important for personal carers to receive this training as they have the greatest amount of contact with care recipients and are well placed to notice changes in their physical and psychological functioning. While it is encouraging that those who work closely with those most in need (high care staff) have better understanding of the depression/physical health link, it would also be useful for low care and community care staff to have improved awareness and knowledge as this may help to prevent care recipients progressing from low care to high care facilities or from the community into residential care.

It is important that cases of depression are identified and that there is optimal evidence-based treatment for both the mental and physical health needs of older persons in care environments. Treatment of physical health issues is essential in reducing the risk for future depression and reducing the risk for greater morbidity and mortality. The need for regular ongoing high quality training to improve knowledge and awareness of this relationship and identification of depression is indicated.

The research has some limitations including the use of non-standardised instruments to measure awareness and knowledge of the depression/physical health link. While it would have been preferable to use a validated instrument none could be found that met the requirements of the current study. The instrument has not been subjected to prior testing and it is therefore uncertain whether responses provide an accurate indication of the underlying construct, that is: awareness of the link between physical morbidity and symptoms of depression. In addition, all the awareness questions are unidirectional which could lead to response biases such as social response bias. The wording of the questions needs further consideration as the use of the words ‘lead to’ (e.g. “to what extent does poorer physical health lead to difficulty sleeping”) may suggest that one causes the other rather than the two being merely associated with each other. Further analysis to establish the validity and reliability of the survey instrument is required. The use of true/false questions may be criticized as a respondent could guess the answers and still get 50% correct. While this is a much used format in surveys and easy for respondents to complete, future research should consider using a different format to measure knowledge. The use of visual analogue scales has been criticized for their highly subjective nature. However they are widely used in psychological research and allow for careful discrimination of values and are a rapid means of gathering ratings [43]. The proportion of professional staff to non-professional staff was very different in the current study with nearly three times as many non-professional staff in the sample. However this does reflect the proportion of these staff populations in the aged care sector. Future studies in this area may consider over-sampling professional staff to equalize the proportions.

In terms of future research, it would be interesting to investigate if there is a relationship between level of staff understanding of the depression/physical health link and actual help-giving behaviours of staff. It would also be useful to explore if depression recognition in care recipients is affected by comorbidity with physical illness and functional disability. Another valuable area of future research would be determining the most appropriate ways to train staff in awareness of the depression/physical health link as those who had had such training in the current study did not have improved understanding.

## Conclusions

Responses to the survey questions demonstrated gaps in knowledge about the relationship between mental and physical health. The need for regular ongoing training to improve knowledge and awareness of this relationship is indicated. Treatment of physical health issues which is essential in reducing the risk for depression in older persons in aged care environments could be optimized by improved staff training.

## Competing interests

The authors declare that they have no competing interests.

## Authors’ contributions

JA contributed to the design of the study and survey instrument, collected survey data, performed the statistical analyses, and wrote the paper. SLN helped with the design of the study and supervision of the research project. GML advised on statistical analysis of the data and assisted with revision of the paper. IBH supervised the research, contributed to the design of the study and assisted with revision of the paper. All authors read and approved the final manuscript.

## Pre-publication history

The pre-publication history for this paper can be accessed here:

http://www.biomedcentral.com/1471-2318/13/30/prepub

## References

[B1] Chapman DP Perry GS Strine TW The vital link between chronic disease and depressive disorders Prev Chronic Dis 2005 2 A14 15670467 PMC1323317

[B2] Australian, Bureau of Statistics Australian Social Trends, 2006: Older people in cared accommodation 2006 Canberra: Australian Bureau of Statistics

[B3] Fleming R Challenging Depression in the elderly: making a determined start 2003 Sydney: University of Sydney

[B4] Beekman AT Deeg DJ van Tilburg T Smit JH Hooijer C van Tilburg W Major and minor depression in later life: a study of prevalence and risk factors J Affect Disord 1995 36 65 75 10.1016/0165-0327(95)00061-5 8988267

[B5] Roberts RE Kaplan GA Shema SJ Strawbridge WJ Does growing old increase the risk for depression? Am J Psychiatry 1997 154 1384 1390 9326820 10.1176/ajp.154.10.1384

[B6] Zheng D Macera CA Croft JB Giles WH Davis D Scott WK Major depression and all-cause mortality among white adults in the United States Ann Epidemiol 1997 7 213 218 10.1016/S1047-2797(97)00014-8 9141645

[B7] Wild B Herzog W Schellberg D Association between the prevalence of depression and age in a large representative German sample of people aged 53 to 80 years Int J Geriatr Psychiatry 2011 27 375 381 21618284 10.1002/gps.2728

[B8] Gurland B The impact of depression on quality of life of the elderly Clin Geriatr Med 1992 8 377 386 1600487

[B9] Gunn JM Ayton DR Densley K The association between chronic illness, multimorbidity and depressive symptoms in an Australian primary care cohort Soc Psychiatry Psychiatr Epidemiol 2012 47 175 184 10.1007/s00127-010-0330-z 21184214

[B10] Celano CM Huffman JC Depression and cardiac disease: a review Cardiol Rev 2011 19 130 142 10.1097/CRD.0b013e31820e8106 21464641

[B11] Pan A Sun Q Okereke OI Depression and risk of stroke morbidity and mortality: a meta-analysis and systematic review JAMA 2011 306 1241 1249 10.1001/jama.2011.1282 21934057 PMC3242806

[B12] Pratt LA Ford DE Crum RM Armenian HK Gallo JJ Eaton WW Depression, psychotropic medication, and risk of myocardial infarction. Prospective data from the Baltimore ECA follow-up. Circulation 1996 94 3123 3129 10.1161/01.CIR.94.12.3123 8989119

[B13] Bair MJ Robinson RL Katon W Depression and pain comorbidity: a literature review Arch Intern Med 2003 163 2433 2445 10.1001/archinte.163.20.2433 14609780

[B14] Druss BG Bradford WD Rosenheck RA Radford MJ Krumholz HM Quality of medical care and excess mortality in older patients with mental disorders Arch Gen Psychiatry 2001 58 565 572 10.1001/archpsyc.58.6.565 11386985

[B15] Stubbs B Zapata-Bravo E Haw C Screening for osteoporosis: a survey of older psychiatric inpatients at a tertiary referral centre Int Psychogeriatr 2009 21 180 186 10.1017/S1041610208007849 18834558

[B16] Blair EW Szarek BL Azhar N Survey of osteoporosis in an inpatient geriatric psychiatric setting: a pilot study Issues Ment Health Nurs 2010 31 403 407 10.3109/01612840903497594 20450342

[B17] Atlantis E Browning C Sims J Diabetes incidence associated with depression and antidepressants in the Melbourne Longitudinal Studies on Healthy Ageing (MELSHA) Int J Geriatr Psychiatry 2010 25 688 696 19806604 10.1002/gps.2409

[B18] Pfaff JJ Draper BM Pirkis JE Medical morbidity and severity of depression in a large primary care sample of older Australians: the DEPS-GP project Med J Aust 2009 190 S75 S80 19351298 10.5694/j.1326-5377.2009.tb02475.x

[B19] Chang-Quan H Xue-Mei Z Bi-Rong D Health status and risk for depression among the elderly: a meta-analysis of published literature Age Ageing 2010 39 23 30 10.1093/ageing/afp187 19903775

[B20] Eisses A Kluiter H Jongenelis K Pot A Beekman A Ormel J Risk indicators of depression in residential homes Int J Geriatr Psychiatry 2004 19 634 640 10.1002/gps.1137 15254919

[B21] Penninx BW Leveille S Ferrucci L van Eijk JT Guralnik JM Exploring the effect of depression on physical disability: longitudinal evidence from the established populations for epidemiologic studies of the elderly Am J Public Health 1999 89 1346 1352 10.2105/AJPH.89.9.1346 10474551 PMC1508750

[B22] Callahan CM Wolinsky FD Stump TE Nienaber NA Hui SL Tierney WM Mortality, symptoms, and functional impairment in late-life depression J Gen Intern Med 1998 13 746 752 10.1046/j.1525-1497.1998.00226.x 9824520 PMC1497028

[B23] Spiegel D Giese-Davis J Depression and cancer: mechanisms and disease progression Biol Psychiatry 2003 54 269 282 10.1016/S0006-3223(03)00566-3 12893103

[B24] Meyer HA Sinnott C Seed PT Depressive symptoms in advanced cancer. Part 2. Depression over time; the role of the palliative care professional Palliat Med 2003 17 604 607 10.1191/0269216303pm813oa 14594151

[B25] Ramasubbu R Patten SB Effect of depression on stroke morbidity and mortality Can J Psychiatry 2003 48 250 257 12776392 10.1177/070674370304800409

[B26] Goldney RD Ruffin R Fisher LJ Asthma symptoms associated with depression and lower quality of life: a population survey Med J Aust 2003 178 437 441 12720509 10.5694/j.1326-5377.2003.tb05408.x

[B27] Peyrot M Rubin RR Levels and risks of depression and anxiety symptomatology among diabetic adults Diabetes Care 1997 20 585 590 10.2337/diacare.20.4.585 9096984

[B28] Romanelli J Fauerbach JA Bush DE The significance of depression in older patients after myocardial infarction J Am Geriatr Soc 2002 50 817 822 10.1046/j.1532-5415.2002.50205.x 12028166

[B29] Kimura M Robinson RG Kosier JT Treatment of cognitive impairment after poststroke depression: a double-blind treatment trial. Stroke 2000 31 1482 1486 10.1161/01.STR.31.7.1482 10884441

[B30] Jorge RE Robinson RG Arndt S Mortality and poststroke depression: a placebo-controlled trial of antidepressants Am J Psychiatry 2003 160 1823 1829 10.1176/appi.ajp.160.10.1823 14514497

[B31] Milani RV Lavie CJ Prevalence and effects of cardiac rehabilitation on depression in the elderly with coronary heart disease Am J Cardiol 1998 81 1233 1236 10.1016/S0002-9149(98)00121-0 9604957

[B32] Barlow JH Turner AP Wright CC A randomized controlled study of the Arthritis Self-Management Programme in the UK Health Educ Res 2000 15 665 680 10.1093/her/15.6.665 11142075

[B33] Lin EH Katon W Von Korff M Effect of improving depression care on pain and functional outcomes among older adults with arthritis: a randomized controlled trial JAMA 2003 290 2428 2429 10.1001/jama.290.18.2428 14612479

[B34] Williams JW Jr Katon W Lin EH The effectiveness of depression care management on diabetes-related outcomes in older patients Ann Intern Med 2004 140 1015 1024 15197019 10.7326/0003-4819-140-12-200406150-00012

[B35] Liu SI Lu RB Lee MB Non-psychiatric physicians’ knowledge, attitudes and behavior toward depression J Formos Med Assoc 2008 107 921 931 10.1016/S0929-6646(09)60015-2 19129052

[B36] Meredith LS Rubenstein LV Rost K Treating depression in staff-model versus network-model managed care organizations J Gen Intern Med 1999 14 39 48 10.1046/j.1525-1497.1999.00279.x 9893090 PMC1496436

[B37] Gallo JJ Meredith LS Gonzales J Do family physicians and internists differ in knowledge, attitudes, and self-reported approaches for depression? Int J Psychiatry Med 2002 32 1 20 10.2190/7QNE-ENF5-2KEL-723X 12075912

[B38] Shao WA Williams JW Jr Lee S Badgett RG Aaronson B Cornell JE Knowledge and attitudes about depression among non-generalists and generalists J Fam Pract 1997 44 161 168 9040519

[B39] Karantzas G Davison T McCabe M Mellor D Beaton P Measuring carers’ knowledge of depression in aged care settings: The Knowledge of Late Life Depression Scale - Revised J Affect Disord 2012 138 417 424 10.1016/j.jad.2012.01.002 22284014

[B40] McCabe MP Russo S Mellor D Effectiveness of a training program for carers to recognize depression among older people Int J Geriatr Psychiatry 2008 23 1290 1296 10.1002/gps.2067 18543348

[B41] McCabe MP Davison T Mellor D George K Knowledge and skills of professional carers working with older people with depression Aging Ment Health 2008 12 228 235 10.1080/13607860701797166 18389403

[B42] Blumenfield M Suojanen J Weiss C Public awareness about the connection between depression and physical health: specifically heart disease Psychiatric Quarterly 2012 83 259 269 10.1007/s11126-011-9199-6 22057371

[B43] Flynn D Schaik P van Wersch A A comparison of multi-item Likert and visual analogue scales for the assessment of transactionally defined coping function Eur J Psychol Assess 2004 20 49 58 10.1027/1015-5759.20.1.49

